# Creation of Low-Loss Triple-Ring Optical Filter via Direct Binary Search Inverse Design

**DOI:** 10.3390/s25185895

**Published:** 2025-09-20

**Authors:** Yuchen Hu, Tong Wang, Wen Zhou, Bo Hu

**Affiliations:** College of Future Information Technology, Fudan University, Shanghai 200438, China; huyuchen@fudan.edu.cn (Y.H.); 24210720274@m.fudan.edu.cn (T.W.)

**Keywords:** coupled mode theory, direct binary search, ring, photonic integration

## Abstract

This paper presents a triple-ring optical filter designed through direct binary search inverse design, comprising three cascaded rings in an add–drop configuration. We established a physical model using temporal coupled-mode theory to derive theoretical spectra and analyze key transmission parameters. Subsequently, we encoded the target transmission performance into a figure of merit to optimize the coupling coefficients between ring resonators and waveguides. We verify the theoretical parameters using three-dimensional finite-difference time-domain simulations. The optimized filter achieves a free spectral range of 86 nm, an insertion loss of 0.4 dB, an extinction ratio of 20 dB, and a narrow spectral linewidth of 0.2 nm within a compact footprint of 29 μm×46.5 μm. This device demonstrates significant application potential, particularly in laser external cavities, dense wavelength division multiplexing systems, and sensing applications. Furthermore, this work provides a systematic design framework for the precision design of photonic devices.

## 1. Introduction

Lasers are the fundamental component of optical communications and photonic integrated circuits (PICs) by delivering high-quality carrier signals [1]. Integrated on-chip external cavity lasers (ECLs) are widely regarded as a transformative technology in the semiconductor industry due to their distinct advantages, such as narrow linewidth, broadening tuning range, and high extinction ratios. The operational bandwidth has been extended from the telecom C-band to the middle-infrared region. A typical on-chip ECL design actively combines a III-V reflective semiconductor optical amplifier (RSOA) gain chip with photonic circuits that including phase shifters, optical filters, and partial-reflection mirrors [2,3,4,5]. The external cavity is typically composed of a ring optical filter formed between the facet of an RSOA and a partial mirror inside a photonic chip. This ring filter not only extends the laser cavity length and narrows the laser output linewidth, but also plays a crucial role in application scenarios such as wavelength selection, channel isolation, and signal modulation [6,7,8]. Conventional single-ring optical filters are widely deployed in optical communication systems owing to high quality factors and compact footprints. Their free spectral range (FSR = λ^2^/(n_g_·*L*)) scales are inverse to its circumference *L*. This relationship limits FSR values in the 1550 nm band to a narrow few-nanometer range, which is insufficient for dense wavelength division-multiplexing (DWDM) broad-tuning requirements [9,10]. Recent advances have demonstrated that cascaded multi-ring optical filters in add–drop configurations leverage the Vernier effect to dramatically enhance FSR, with theoretical analysis showing that their tuning range surpasses conventional single-ring optical filters by multiple orders of magnitude [11], which establishes this architecture as a promising approach for developing high-performance optical filters. In 2022, Guo et al. [12] introduced a novel dual-ring cascaded structure leveraging the Vernier effect. This configuration comprises a cascaded add–drop racetrack micro-ring resonator (MRR) featuring precisely controlled perimeter variations in circumference *L*. Through rigorous numerical simulations using the finite-difference time-domain (FDTD) method, the designed filter demonstrated an extended FSR of 95 nm within the C-band (centered at 1550 nm). However, the footprint is relatively large (~8 mm total length), and the insertion loss varies by 1.5 dB in the 1480–1660 nm wavelength range, which may constrain its application in PIC. In 2023, Calo et al. [13] developed a hybrid InP-SiN multi-ring filter that leverages SiN waveguides with low propagation losses (0.1 dB/cm). This design capitalizes on the material’s moderate refractive index contrast to achieve a smaller bending radius. While the researchers constructed a relatively compact device and meticulously calibrated the power coupling coefficient of the MRR. However, the resulting FSR remains limited to 45 nm. In 2024, Lin et al. [14] reported a novel Vernier cascaded dual-ring filtering structure with ultrabroadband operation capability. This configuration incorporates two serially connected add–drop MRRs featuring a marginally distinct radius. Parameter optimization was achieved through finite-difference eigenmode simulations in the Lumerical environment, complemented by dispersion characteristics analysis of Si_3_N_4_-based MRRs. Simulation results revealed that the optimized device exhibits an insertion loss of 1 dB, an extinction ratio of 5.78 dB, and an FSR reaching 72 nm. While the demonstrated extinction ratio exceeds the 5 dB threshold, it is sufficient for lasing, and its implementation in DWDM systems may induce substantial crosstalk due to this parameter limitation. Furthermore, the designed structure requires refinement for better transmission performance.

Despite researchers having made significant progress in the study of multi-ring optical filters, current design methodologies still fundamentally rely on experimental iteration [15,16], which means that the structural characteristics are manually optimized through iterative processes to satisfy key optical performance parameters, including transmission efficiency, FSR, and extinction ratios. These approaches account for only a limited range of structural parameters, neglecting coupling losses between ring resonators and waveguides, which often converge to local optima and fail for high precision and densely integrated devices. As a gradient-free optimization algorithm, direct binary search (DBS) provides a potential solution to these issues. The core concept of this methodology involves transforming the parameter space into a discrete format via binary encoding, while employing an iterative strategy of sequentially perturbing individual parameters to quantify the extent of performance optimization. This DBS algorithm effectively avoids local optima traps while maintaining dual compatibility with both discrete and continuous variables. In recent years, DBS has been effectively applied in the development of modern silicon photonic devices. In 2023, Lin et al. [17] introduced a low-loss, broadband polarization-insensitive high-order mode pass filter optimized using the DBS algorithm. The device’s performance was modeled through 3D-FDTD numerical simulations, and the DBS framework was applied to refine the coupling architecture between multi-mode waveguides. Simulation results demonstrated that within the 1520–1590 nm wavelength range, the filter achieved insertion losses below 0.86 dB and extinction ratios exceeding 16.8 dB for TE polarization. For TM polarization, the performance improved further, with insertion losses reduced to under 0.79 dB and extinction ratios surpassing 17.5 dB. In 2024, Wang et al. [18] proposed an 1×2 ultra-compact $2.4\times 3.6\;{\mu m}^{2}$ multimode wavelength demultiplexer utilizing the DBS algorithm, and demonstrated simultaneous wavelength separation and mode transformation capabilities. The optimization process was driven by predefined figure-of-merit (FOM) parameters that guided the algorithm in enhancing device transmittance. Simulation results revealed minimum insertion loss and extinction ratio values of 0.759 dB and 10.06 dB, respectively. Concurrently, Zhou’s team [19] developed a 1×2 photonic switching device on a silicon-on-insulator platform assisted with a DBS algorithm. Through systematic parameter optimization, this compact design achieved a remarkably small footprint of $3\times 4\;{\mu m}^{2}$ while maintaining insertion loss below 0.5 dB and exceeding 20 dB extinction ratio performance. Recent reports [20,21] indicate that designed photonic devices assisted by the DBS algorithm can achieve compact footprints while maintaining low loss through exceptional optimization of their coupling structures. However, not a lot of research has been conducted to date regarding the application of a DBS algorithm in Multi-ring optical filter design, primarily due to the significantly increased complexity arising from multiple coupled resonators. Applying DBS to such structures introduces unique challenges, including a high-dimensional parameter space for optimization, the formulation of effective FOM for the algorithm, and trade-offs among performance metrics such as insertion loss and linewidth. The coupling between the ring and the waveguide is the core of the multi-ring filter device. To efficiently implement the DBS algorithm in such optical filtering configurations, conducting a thorough systematic analysis of the MRR coupling architectures becomes essential. Commonly, the transmission characteristics of this coupling structure can be analyzed by using the time-domain coupled-mode theory (TCMT). Hence, there is considerable interest in evaluating the transmission efficiency of the coupling structure based on TCMT and designing a multi-ring optical filter with excellent performance and compact structures assisted by the DBS algorithm.

This paper presents a low-loss triple-ring optical filter designed using TCMT and the DBS algorithm. We first developed a TCMT-based physical model for a cascaded triple-ring system. Subsequently, we encoded the target transmission characteristics into an FOM and employed the DBS algorithm to optimize the coupling coefficients between the rings and waveguides. We then validated the theoretical parameters through 3D-FDTD simulations. The optimized filter achieves an insertion loss of 0.4 dB, an FSR of 86 nm, a linewidth of 0.2 nm, and an extinction ratio of 20 dB. This device offers substantial benefits for applications in ECL and DWDM systems. Furthermore, our study establishes a novel theoretical framework and technical approach for designing high-precision multi-ring optical filters.

## 2. Theoretical Analysis

### 2.1. Theoretical Analysis of the Triple-Ring Optical Filter

**Figure 1:**
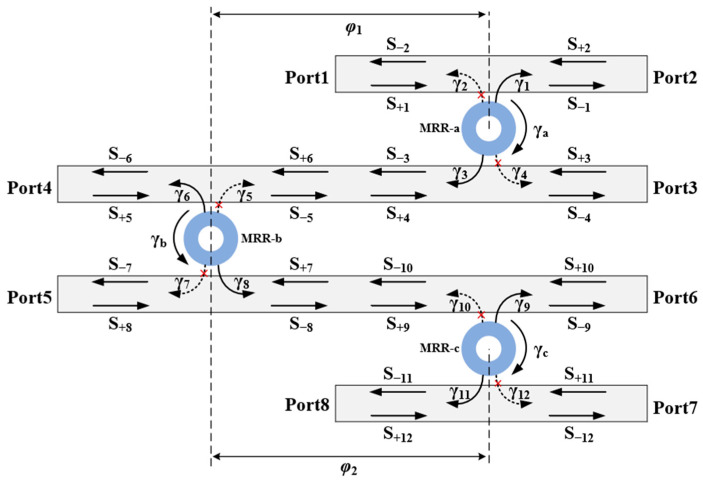
Theoretical model of triple-ring optical filter.

The filter structure proposed in this study comprises three MRRs and four waveguides in cascade. The physical model for a triple-ring optical filter is shown in Figure 1. In the cascade triple-ring system, the coupling process occurs as follows: when the wavelength of light incident from Port 1 coincides with the resonant wavelengths of both MRR-a, MRR-b, and MRR-c, the optical wave sequentially couples into all MRRs. Eventually, most of the light intensity is output from Port 8, while all other ports exhibit negligible energy output. In Figure 1, $S_{+i}$ and $S_{-i}$ represent the amplitude of the input and output waves, respectively (i = 1, 2, 3… 12). $\varphi_{1}$ and $\varphi_{2}$ represent the phase delay from MRR-a to MRR-b and from MRR-b to MRR-c, respectively, determined by the distance between the ring cavities. $\gamma_{i}$ (*i* = 1, 2, 3…12) are the amplitude coupling attenuation coefficients from the rings to the waveguides. Due to the unidirectional propagation of light energy within the ring resonator, traveling wave resonance is established through periodic circulation. Consequently, there is no backward coupling, i.e., $\gamma_{2}=\gamma_{4}=\gamma_{5}=\gamma_{7}=\gamma_{10}=\gamma_{12}=0$. $\gamma_{a}$, $\gamma_{b}$, and $\gamma_{c}$ denote the amplitude intrinsic attenuation coefficients for MRR-a, MRR-b, and MRR-c, respectively. The intrinsic quality factor $Q_{\mathrm{int}}$ is related to the intrinsic loss rate α through the expression $Q_{\mathrm{int}}=\frac{2\pi n_{\mathrm{eff}}L}{\lambda }\cdot \frac{v_{g}}{\alpha }$ where L denotes the optical path length, $V_{g}$ represents the group velocity of the light, λ is the wavelength, and $n_{\mathrm{eff}}$ is the effective refractive index of the medium. The smaller $\gamma_{a}$, $\gamma_{b}$ or $\gamma_{c}$ (reduced α), the higher the intrinsic quality factor $Q_{\mathrm{int}}$. Due to the radius mismatch between the three rings, we define the resonant frequencies as $\omega_{1}$, $\omega_{2}$ and $\omega_{3}$, the resonant mode amplitudes as *a, b,* and *c* for MRR-a, MRR-b, and MRR-c, respectively. Let the incident light frequency be ω. The temporal coupled-mode equations for this structure are then given by the following:

For MRR-a, the time domain variation in the resonant mode amplitude can be expressed as follows:

(1) $$\frac{da}{dt}=-i\omega_{1}a-(\gamma_{1}+\gamma_{3}+\gamma_{a})a+\sqrt{2\gamma_{1}}S_{+1}+\sqrt{2\gamma_{3}}S_{+3}$$

According to power conservation and time-reversal symmetry, the relationship between input and output waves is given as follows:

(2) $$S_{-1}=S_{+1}-\sqrt{2\gamma_{1}}\cdot a$$

(3) $$S_{-3}=S_{+3}-\sqrt{2\gamma_{3}}\cdot a$$

For MRR-b, the time domain variation in the resonant mode amplitude is given by the following:

(4) $$\frac{db}{dt}=-i\omega_{2}b-(\gamma_{6}+\gamma_{8}+\gamma_{b})b+\sqrt{2\gamma_{6}}S_{+6}+\sqrt{2\gamma_{8}}S_{+8}$$

Similarly, the relationship between input and output waves is given as follows:

(5) $$S_{+6}=S_{-3}\cdot e^{j\varphi_{1}}$$

(6) $$S_{-6}=S_{+6}-\sqrt{2\gamma_{6}}\cdot b$$

(7) $$S_{-8}=S_{+8}-\sqrt{2\gamma_{8}}\cdot b$$

For MRR-c, the time domain variation in the resonant mode amplitude can be expressed as follows:

(8) $$\frac{dc}{dt}=-i\omega_{3}c-(\gamma_{9}+\gamma_{11}+\gamma_{c})c+\sqrt{2\gamma_{9}}S_{+9}+\sqrt{2\gamma_{11}}S_{+11}$$

Similarly, the relationship between input and output waves is given as follows:

(9) $$S_{+9}=S_{-8}\cdot e^{j\varphi_{2}}$$

(10) $$S_{-9}=S_{+9}-\sqrt{2\gamma_{9}}\cdot c$$

(11) $$S_{-11}=S_{+11}-\sqrt{2\gamma_{11}}\cdot c$$

Assuming that $S_{+2}=S_{+3}=S_{+5}=S_{+8}=S_{+10}=S_{+11}=S_{+12}=0$, which means that there is no light input at Ports 2, 3, 4, 5, 6, 7, and 8. Since the light waves are only input from Port 1, by combining Equations (1)–(11), we can obtain the transmission spectrum T(ω) of the cascaded triple-ring system:

(12) $$T(\omega )={\left|\frac{S_{-11}}{S_{+1}}\right|}^{2}=\frac{64\gamma_{1}\gamma_{3}\gamma_{6}\gamma_{8}\gamma_{9}\gamma_{11}e^{j2(\phi_{1}+\phi_{2})}}{\left[{\left(\gamma_{1}+\gamma_{3}+\gamma_{a}\right)}^{2}+{\left(\omega -\omega_{1}\right)}^{2}\right]\left[{\left(\gamma_{6}+\gamma_{8}+\gamma_{b}\right)}^{2}+{\left(\omega -\omega_{2}\right)}^{2}\right]\left[{\left(\gamma_{9}+\gamma_{11}+\gamma_{c}\right)}^{2}+{\left(\omega -\omega_{3}\right)}^{2}\right]}$$

For the MRR, the maximum coupling efficiency can be obtained when the MRR reaches the critical coupling point [22]. At critical coupling, the MRR mode loses the same power to the internal dissipation mechanisms as to the coupling to the waveguide mode [23]. The expression of the critical coupling for the three MRRs is as follows:

(13) $$\gamma_{1}=\gamma_{3}+\gamma_{a}$$

(14) $$\gamma_{6}=\gamma_{8}+\gamma_{b}$$

(15) $$\gamma_{9}=\gamma_{11}+\gamma_{c}$$

assuming the three MRRs with no frequency detuning ($\omega_{1}=\omega_{2}=\omega_{3}=\omega_{0}$). Substituting Equations (13)–(15) into Equation (12), the maximum transmittance $T_{\max}$ of the cascaded triple-ring system can be expressed as follows:

(16) $$T_{\max}={\left|\frac{S_{-11}}{S_{+1}}\right|}^{2}=\frac{(\gamma_{1}-\gamma_{a})(\gamma_{6}-\gamma_{b})(\gamma_{9}-\gamma_{c})e^{j2(\varphi_{1}+\varphi_{2})}}{\gamma_{1}\gamma_{6}\gamma_{9}}$$

From Equation (16), it can be seen that $T_{\max}$ can reach 1 only when $\varphi_{1}+\varphi_{2}=(m+1/2)\pi$ (where m is a non-negative integer) and $\gamma_{a}=\gamma_{b}=\gamma_{c}=0$; furthermore, the maximum transmittance is significantly influenced by the coupling attenuation coefficient ($\gamma_{1}$, $\gamma_{3}$, $\gamma_{6}$, $\gamma_{8}$, $\gamma_{9}$, and $\gamma_{11}$) between MRRs and waveguides. This implies that the coupling structures on both sides of each ring critically govern the overall transmission efficiency of the cascaded triple-ring system.

### 2.2. Analysis of the Frequency Deviation

Based on the above theoretical derivation results, we further analyze the relationship between the transmittance and frequency detuning. Assuming a symmetric coupling structure between the MRRs and waveguides, we set equal coupling attenuation coefficients $\gamma_{1}=\gamma_{3}=\gamma_{6}=\gamma_{8}=\gamma_{9}=\gamma_{11}=\gamma_{\mathrm{wav}}$. Let $a=x_{1}$, $b=x_{2}$, $c=x_{3}$. According to Equation (15), the transmission spectrum T(ω) of cascaded triple-ring system can be rewritten as follows:

(17) $$T(\omega )={\left|\frac{S_{-11}}{S_{+1}}\right|}^{2}=\frac{{\left(4\gamma_{\mathrm{wav}}^{2}\right)}^{3}e^{j2(\varphi_{1}+\varphi_{2})}}{\prod_{i=1}^{3}\left[{\left(2\gamma_{\mathrm{wav}}+\gamma_{x_{i}}\right)}^{2}+{\left(\omega -\omega_{i}\right)}^{2}\right]}$$

where $\omega_{i}$, (*i* = *a*,*b*,*c*) and $\gamma_{x_{i}}$, (*i* = *a*,*b*,*c*) denote the resonant frequencies and the intrinsic amplitude attenuation coefficients of MRR-a, MRR-b, and MRR-c, respectively. Modeling the MRR as lossless resonators, that is, $\gamma_{x_{i}}=0$, (*i* = *a*,*b*,*c*). Let $\omega -\omega_{0}$ be the frequency detuning with identical resonant frequencies $\omega_{1}=\omega_{2}=\omega_{3}=\omega_{0}$. Equation (17) can be simplified as follows:

(18) $$T(\omega )={\left|\frac{S_{-11}}{S_{+1}}\right|}^{2}=\frac{{\left(4\gamma_{\mathrm{wav}}^{2}\right)}^{3}e^{j2(\varphi_{1}+\varphi_{2})}}{{\left[{\left(2\gamma_{\mathrm{wav}}\right)}^{2}+i{\left(\omega -\omega_{0}\right)}^{2}\right]}^{3}}$$

**Figure 2:**
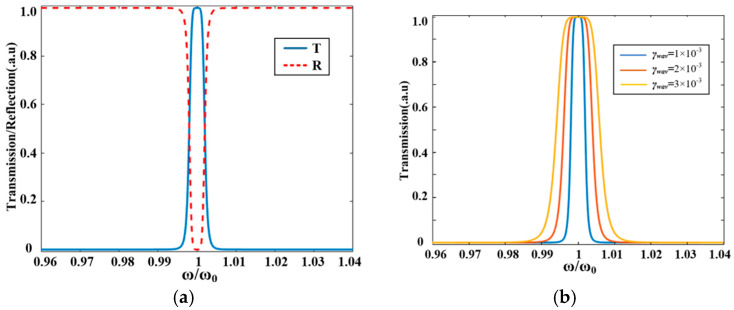
The relationship between transmittance and frequency detuning of the triple-ring cascaded system: (**a**) spectrum of transmission and reflection; (**b**) different amplitude attenuation coefficients.

Equation (18) demonstrates that the transmission T(ω)=1 when $\varphi_{1}=\varphi_{2}=(m+1/2)\pi$, (where *m* is a non-negative integer) and $\omega =\omega_{0}$. Figure 2 plots the transmittance versus frequency detuning in the triple-ring cascaded system. As shown in Figure 2a, the transmittance peaks while the reflectance minimizes at the resonant frequency $\omega =\omega_{0}$. Increasing frequency detuning ($\omega /\omega_{0}$) reduces transmittance and elevates reflectance, consistent with energy conservation principles. Figure 2b further demonstrates progressive broadening of the transmission spectrum with rising coupling attenuation coefficient at $\gamma_{\mathrm{wav}}$ = $1\times 10^{-3}$, $2\times 10^{-3}$, and $3\times 10^{-3}$. The total Q-factor ($Q_{\mathrm{total}}$) of the MRR is governed by its intrinsic Q-factor ($Q_{i}$) and coupling Q-factor ($Q_{c}$) components, related through $Q_{\mathrm{total}}=\frac{1}{Q_{i}}+\frac{1}{Q_{c}}$. Enhanced waveguide coupling strength (indicated by rising $\gamma_{\mathrm{wav}}$) facilitates greater optical energy exchange between the waveguide and MRR. This elevates $Q_{c}$, thereby reducing $Q_{\mathrm{total}}$ and broadening the linewidth.

Conclusively, designing a low-loss narrow-linewidth triple-ring optical filter requires three core conditions: (1) The phase delay matching $\varphi_{1}=\varphi_{2}=(m+1/2)\pi$ (where *m* is a non-negative integer) between adjacent MRRs; (2) The three MRRs must resonate without frequency detuning ($\omega_{1}=\omega_{2}=\omega_{3}=\omega_{0}$); and (3) minimized intrinsic loss $\gamma_{x_{i}}$, (*i* = *a*,*b*,*c*). Simultaneously, Figure 2b reveals that maintaining low $\gamma_{\mathrm{wav}}$ values ensures a narrow transmission linewidth. The theoretical results above will be validated by using FDTD simulations with the DBS algorithm.

## 3. Device Design

### 3.1. The Working Principle of DBS

**Figure 3:**
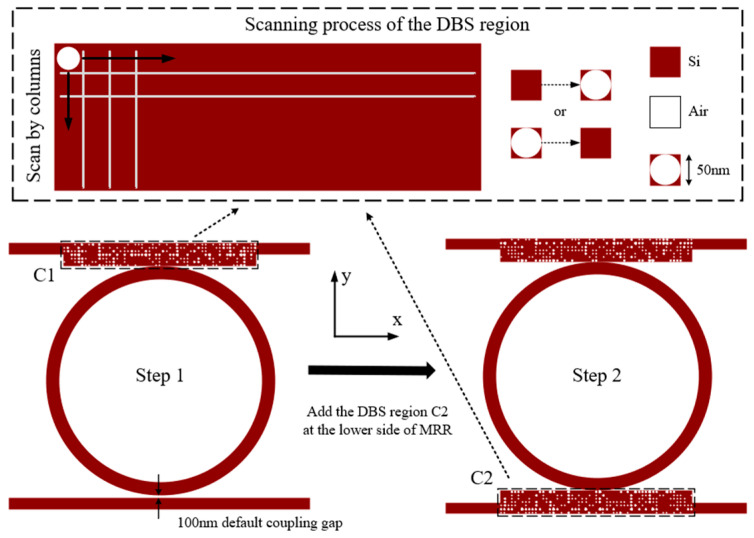
The workflow of DBS inverse design.

This study applies DBS inverse design to optimize the coupling configurations. To accelerate the preliminary construction of the device model, we initially employed a sequential optimization process by maintaining a default 100 nm coupling gap for the lower region. Figure 3 presents the DBS-based inverse design workflow for coupling structures. After completing the upper region scan, we incorporate the lower region into the DBS design domain for subsequent scanning. The optimization initiates by solving electromagnetic equations for the initial structure (full-silicon state in Figure 3) and calculating the FOM using transmittance at Port 8 and reflectance at Port 1. The algorithm then scans column wise: it modifies the state of the first row, first column pixel from silicon to air hole embedded, recomputes electromagnetic field distributions, and updates the FOM. By comparing the new FOM with the previous value, it retains the modification if FOM increases; otherwise, it reverts the pixel state. This process iterates sequentially through all pixels (e.g., first row, second column, and next) until completing the entire design region scan.

There are two monitors placed in the designed triple-ring cascaded structure at Port 8 and Port 1 for transmittance (*T*) and reflectance (*R*) detection. To dynamically evaluate device performance during inverse design, we introduce a FOM defined as follows:

(19) FOM=T−κ⋅R

Here, *T* denotes peak transmittance, *R* represents peak reflectance, and κ serves as the reflectance penalty coefficient. Under ideal scattering-free conditions, energy conservation dictates T+R=1. In practical systems, however, optical transmission losses reduce this sum to T+R<1. Defining FOM directly as T−R would yield identical values for divergent performance states, preventing convergence in inverse design due to ambiguous optimization direction. This necessitates introducing coefficient κ, which quantifies the transmittance compensation (κ%) required to offset each 1% reflectance increase, essentially weighting *R*’s impact (we set κ = 2). Consequently, the meaning represented by Equation (19) is to first ensure a high transmittance *T*, and then to reduce the impact of *R* on the transmission loss during inverse design. Guided by this principle, the DBS inverse design optimizes the coupling structure of the triple-ring optical filter. During each iteration, our algorithm preserves structural changes when the FOM increases and advances the optimization. If the FOM decreases, the algorithm instantly reverts to the previous structure and tests the next pixel. After establishing the upper and lower coupling regions for each MRR, we perform random sampling scans on the six DBS regions for global optimizations. The pseudocode flowchart is shown in Algorithm 1.
**Algorithm 1** Direct Binary Search Method for Multi-Ring Filter Design**Input:**(1)$\mathrm{The}\;\mathrm{maximum}\;\mathrm{number}\;\mathrm{of}\;\mathrm{DBS}\;\mathrm{regions}\;C_{\max}$(2)(i×j) Grid parameters (7×55, spacing 150 nm)(3)Cylinder parameters (radius 50 nm, height 220 nm)(4)Simulation wavelength range: (1480–1650 nm)(5)$\mathrm{The}\;\mathrm{maximum}\;\mathrm{number}\;\mathrm{of}\;\mathrm{epochs}\;E_{\max}$(6)The reflectance penalty coefficient κ 
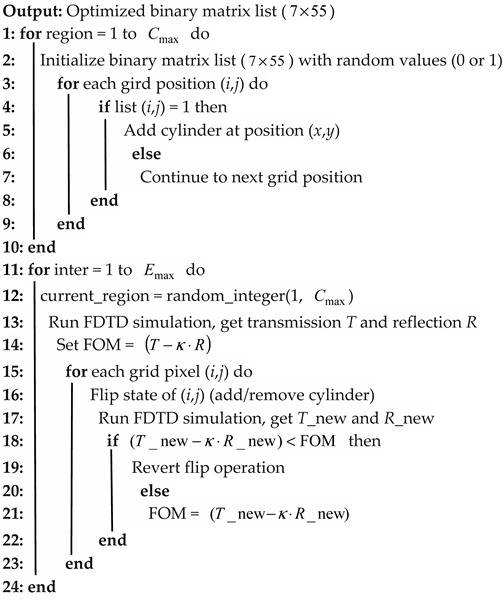


### 3.2. Design of the Triple-Ring Optical Filter

**Figure 4:**
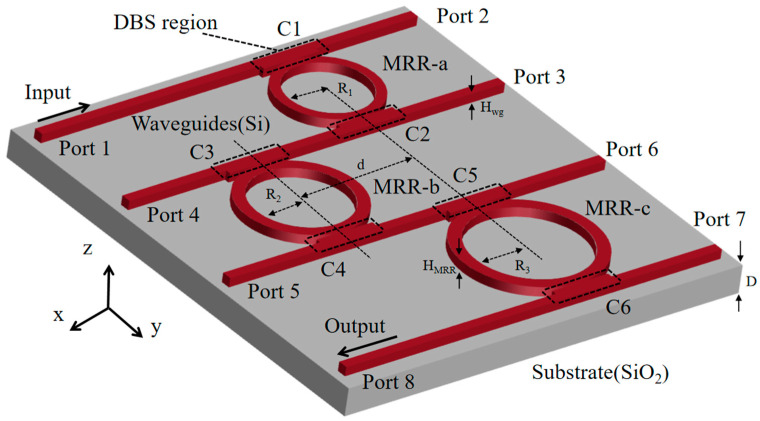
Triple-ring optical filter structure diagram.

Figure 4 presents the diagram of the triple-ring optical filter structure designed in this paper. The cascaded structure was constructed using the FDTD module in commercial software Lumerical 2024 R1, comprising three rings with a radius $R_{1}$, $R_{2}$, and $R_{3}$ of 5 μm, 5.9 μm, and 9.7 μm, respectively, along with four waveguides. All rings and waveguides share identical dimensions of 0.45 μm width, 0.22 μm thickness, and silicon material. The SiO_2_ substrate has 4 μm thickness, and the spacing between the rings is 13 μm. The structural parameters are summarized in Table 1.

**Figure 5:**
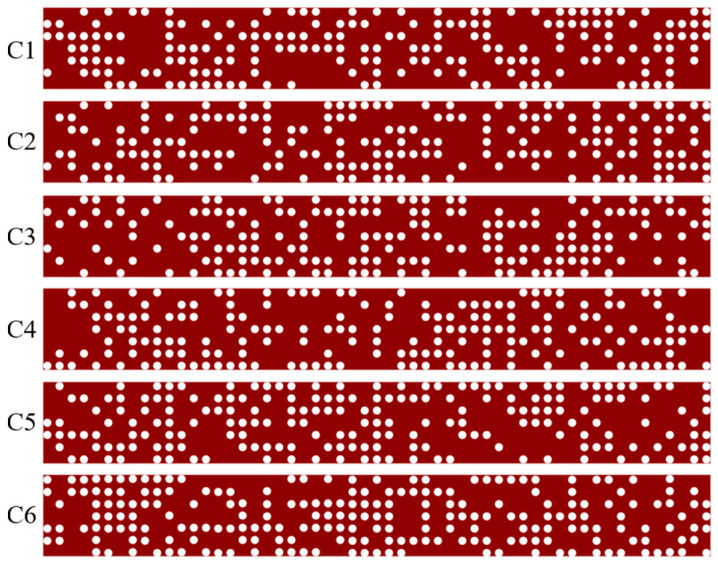
Structural configurations of the six DBS regions.

Figure 4 further reveals eight ports in the designed structure. We position the light source at Port 1, deploy a reflectance monitor at Port 1, and place a transmittance monitor at Port 8. Section 2 analysis of Equation (16) indicates that the coupling strength between each ring and waveguide directly governs the transmission efficiency of the cascaded system. Consequently, we implement six DBS design regions (labeled C1–C6 in Figure 4) across the upper and lower sides of MRR-a, MRR-b, and MRR-c. Each region comprises an 8.2 μm  ×  1 μm silicon slab patterned with 7×55 cylindrical pixels (0.05 μm diameter) arranged in a square lattice photonic crystal configuration. The binary encoding system actively assigns two operational states (0 and 1) to each pixel. When the algorithm sets a pixel to State 1, it constructs an air-filled cylindrical structure with a 0.22 μm height and renders this state white in visualizations. Conversely, activating State 0 completely fills the pixel with solid silicon material and displays it in red. Figure 5 illustrates the structural configurations of the six DBS regions in the triple-ring optical filter achieved through the DBS algorithm.

## 4. Numerical Calculations

### 4.1. Optimization Based on DBS

**Figure 6:**
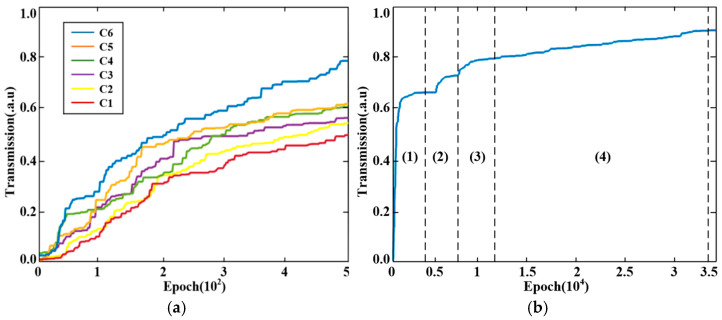
Transmittance variation with the number of iterations: (**a**) Independent optimizations; and (**b**) Global optimizations.

Figure 6 plots the transmittance evolution throughout the inverse design iterations. Figure 6a specifically tracks independent optimizations of structures C1 to C6. As previously established, we maintain a default 100 nm coupling gap for the lower coupling structure during the upper region optimization to accelerate convergence. After completing the upper region scan, we incorporate the lower DBS design domain for subsequent scanning. Consequently, Figure 6a reveals that each new DBS region initiates transmittance growth from zero. This occurs because every added region starts iterations on a 7×55 random matrix where stochastically distributed air holes cannot guarantee optimal coupling. The plot further demonstrates progressive transmittance enhancement as new regions integrate into the design. This improvement stems from building upon previously optimized structures, systematically pushing performance boundaries. After 500 independent iterations, the maximum transmittance values for C1, C2, C3, C4, C5, and C6 can reach 0.49, 0.51, 0.52, 0.6, 0.62, and 0.78, respectively. This partitioned optimization strategy rapidly achieves high transmittance while constructing the initial device framework, significantly boosting design efficiency.

We perform approximately 35,000 iterations on the triple-ring filter for global optimizations, with peak transmittance values documented in Figure 6b. The initial transmittance starts at zero because we simultaneously incorporate all six DBS regions into the simulation domain during global optimization in Figure 6b, rather than conducting separate simulations per region as in Figure 6a. This integrated approach enables comprehensive analysis of transmission efficiency across the triple-ring cascaded system. The plot clearly delineates four distinct optimization phases labeled (1)–(4), where Phase (1) involves approximately 4000 iterations optimizing structures C1 and C2 on MRR-a until transmittance plateaus at 0.62, after which we save the configuration and commence Phase (2) to optimize C3 and C4 on MRR-b through another 4000 iterations until performance stabilizes at 0.72 transmittance. Consequently, we preserve this structure and initiate Phase (3), building upon Phase (2)’s structure to optimize C5 and C6 on MRR-c via 4000 additional iterations, observing diminishing returns as transmittance exceeds 0.81. This progression demonstrates that Phases (1)–(3) substantially enhance maximum transmittance beyond Figure 6a’s independent optimizations through intensified iteration depth. At approximately the 12,000th iteration, we enter Phase (4), performing random sampling scans across all six DBS regions within the saved triple-ring structure from Phase (3), with Figure 6b revealing stabilized peak transmittance at 0.91 between iterations 12,000 and 34,000. Finally, we conduct 1000 iterations to ensure that the transmittance will not change any further, completing the inverse design process after approximately 35,000 total iterations.

### 4.2. Transmission Spectra

**Figure 7:**
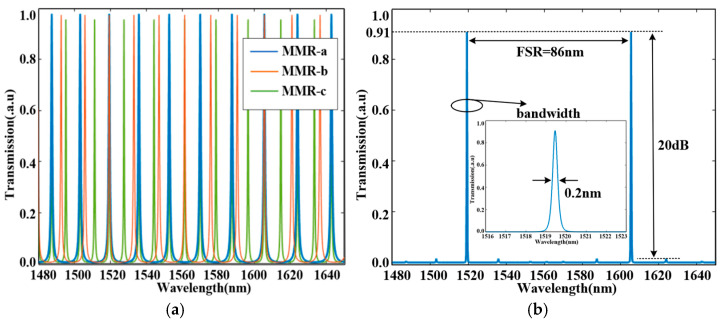
Transmission spectra at the wavelength range from 1480 nm to 1650 nm: (**a**) Comparison of resonance peaks; (**b**) Transmission spectra of the proposed device.

Based on the structure optimized by the DBS algorithm, we perform FDTD simulations to characterize the triple-ring optical filter’s transmission spectrum across 1480 nm to 1650 nm operational wavelengths. Figure 7a displays individual transmission spectra for the three MRRs: blue, orange, and green lines represent MRR-a, MRR-b, and MRR-c, respectively. Slight differences in circumferences between the rings produce distinct FSRs, leading to coincident resonant peaks at 1519.5 nm and 1605.5 nm through the Vernier effect, while maintaining offsets at other wavelengths. As shown in Figure 7a, the peak transmittance values of MRR-a, MRR-b, and MRR-c are approximately 0.98, 0.98, and 0.96, respectively. The lower peak transmittance of MRR-c is due to its larger circumference, which leads to higher propagation loss. The estimated losses of the three MRRs are approximately 0.08 dB for MRR-a, 0.08 dB for MRR-b, and 0.18 dB for MRR-c. An additional loss of about 0.06 dB originates from the vertical direction of the device. The transmission spectrum of the proposed device is shown in Figure 7b, demonstrating a peak transmittance of 0.91, which corresponds to an insertion loss of 0.4 dB, along with an extinction ratio of 20 dB, a FSR of 86 nm, and a linewidth of 0.2 nm.

**Figure 8:**
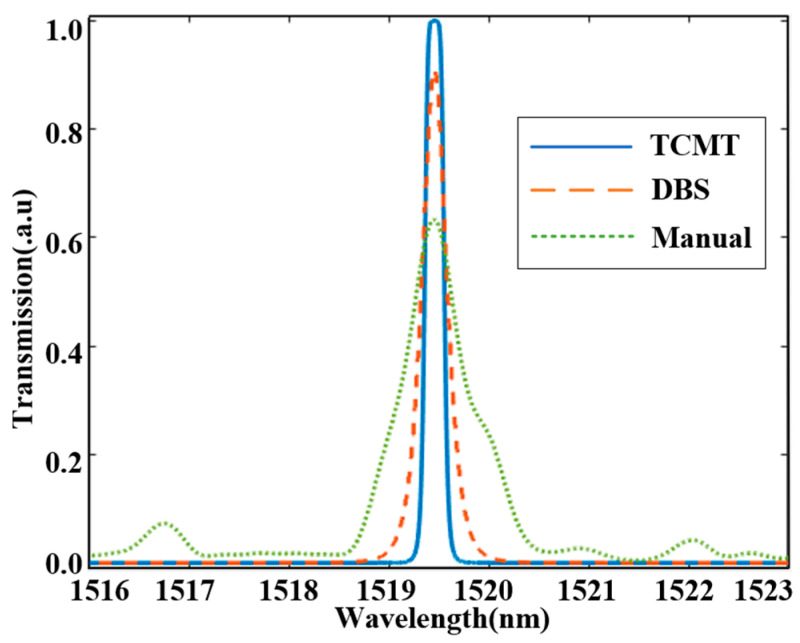
Comparison of the theoretical and simulated transmission spectra.

The transmission spectra comparison between TCMT and the DBS algorithm is shown in Figure 8. The blue curve plots the theoretical spectrum calculated via TCMT Equation (18), contrasting with the orange dashed line showing FDTD-simulated results using the DBS algorithm. The green dotted line depicts conventional manual design. At the operational wavelength of 1519.5 nm, peak transmittance values of TCMT, DBS, and Manual are 1, 0.91, and 0.62, respectively. Two primary factors account for the theoretical maximum transmittance surpassing FDTD-simulated results. First, to simplify the derivation process of theoretical transmission spectra, we intentionally neglect intrinsic loss ($\gamma_{x_{i}}=0$, *i* = *a*,*b*,*c*) when modeling MRR-a, MRR-b, and MRR-c. Second, the FDTD simulation method incorporates broader loss mechanisms compared to the TCMT equations, comprehensively accounting for waveguide propagation loss, MRR’s bending loss, and vertical (z-axis) structural losses across the entire device. Furthermore, the trend observed in the theoretical transmission spectrum derived based on TCMT is basically consistent with that calculated by the DBS algorithm, while the transmission efficiency of the devices designed manually is significantly lower.

### 4.3. Steady-State Field Distribution

**Figure 9:**
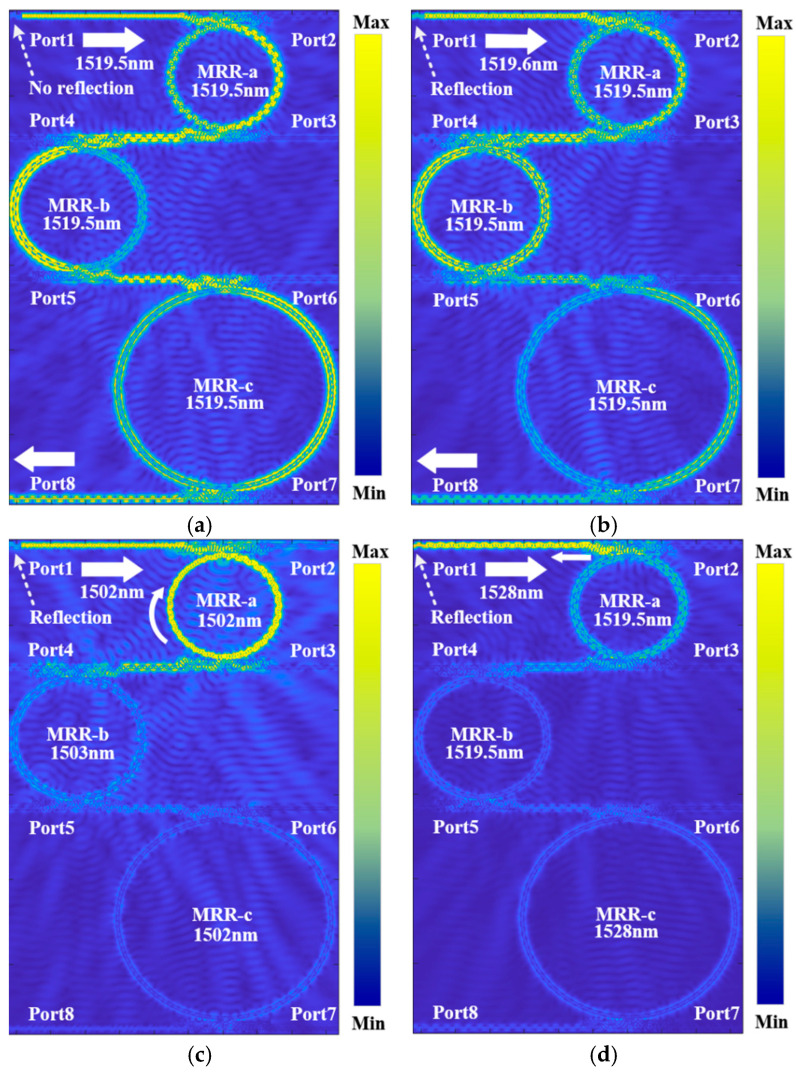
Steady-state field distribution at different operating wavelengths: (**a**) 1519.5 nm; (**b**) 1519.6 nm; (**c**) 1502 nm; and (**d**) 1528 nm.

Figure 9a–d display the steady-state field distributions for input wavelengths of 1519.5 nm, 1519.6 nm, 1502 nm, and 1528 nm incident from Port 1. As established previously, the resonant peaks of MRR-a, MRR-b, and MRR-c coincide at 1519.5 nm. Accordingly, Figure 9a shows efficient sequential coupling of the input light through all three rings, resulting in high transmission at Port 8 and minimal back coupling to Port 1 as mentioned in Section 2. In Figure 9b, a slight wavelength shift of 0.1 nm causes an increase in the light intensity at Port 1. The resulting wavelength mismatch leads to partial coupling and the formation of a standing wave pattern near the MRR-a upper-coupling region, indicative of interferometric cancelation between forward and backward propagating waves. This is accompanied by increased energy reflection detectable at Port 1. At the more significantly detuned wavelength of 1502 nm, as shown in Figure 9c, MRR-a and MRR-c are near resonance, whereas MRR-b is off resonance. Most light couples into MRR-a and transfers to the intermediate bus waveguide, but is largely reflected before reaching Port 8 due to the spectral misalignment with MRR-b, again manifesting as a standing wave pattern in the bus waveguide adjacent to MRR-b. Finally, at 1528 nm, as shown in Figure 9d, where no ring is resonant, strong reflection occurs at the MRR-a. The field plot clearly shows a high light intensity at Port 1, resulting from the constructive interference of the incident and reflected waves.

### 4.4. Fabrication Tolerance of the Device

**Figure 10:**
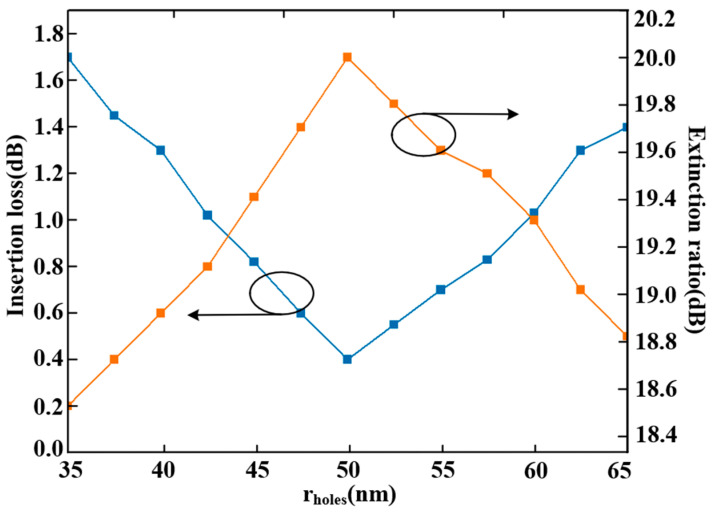
Insertion loss and Extinction ratio versus radius of air holes.

**Figure 11:**
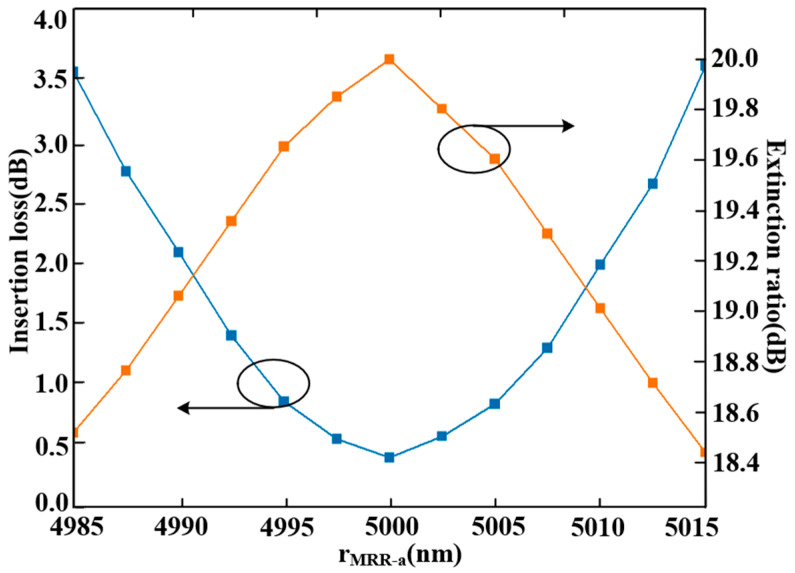
Insertion Loss and Extinction Ratio versus Radius of MRR-a.

**Figure 12:**
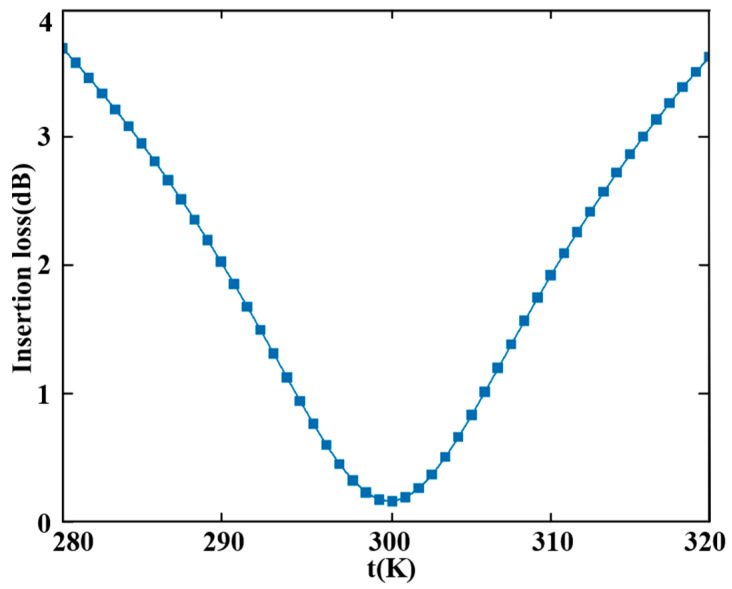
The relationship between Insertion Loss and temperature t.

The practical deployment of photonic devices necessitates rigorous analysis of fabrication tolerances and thermal stability. In our triple-ring filter, performance is particularly sensitive to dimensional variations in the air holes ($r_{\mathrm{holes}}$) of the coupling structure and the radius of the rings ($r_{\mathrm{MRR}}$). Furthermore, insertion loss is susceptible to fluctuations in operating temperature (t). This section systematically investigates the individual impact of these fabrication and thermal variations on device performance:

(1) The radius of the air holes ($r_{\mathrm{holes}}$) in the coupling structure was set to 50 nm. To evaluate fabrication tolerance, we varied $r_{\mathrm{holes}}$ and analyzed its impact on device performance. As shown in Figure 10, both the insertion loss and extinction ratio are highly sensitive to variations in $r_{\mathrm{holes}}$. A transmittance of ≥80% is achieved when the radius ranges from 42.8 nm to 58.2 nm. Beyond this range, the transmission efficiency decreases due to the weak coupling coefficient between the rings and waveguides. The insertion loss reaches its minimum at the designed value of $r_{\mathrm{holes}}=50\;\mathrm{nm}$. Thus, the allowable fabrication tolerance for the air hole diameter is approximately ±7.2 nm, corresponding to a total diameter variation range of 30.8 nm.
(2) The designed radii for the three MRRs in the proposed triple-ring filter are $r_{\mathrm{MRR}-a}=5000\;\mathrm{nm}$, $r_{\mathrm{MRR}-b}=5900\;\mathrm{nm}$, and $r_{\mathrm{MRR}-c}=9700\;\mathrm{nm}$. Altering any of these radii leads to resonant wavelength mismatch, thereby degrading device performance. To quantify this effect, we varied $r_{\mathrm{MRR}-a}$ and examined the corresponding response. As illustrated in Figure 11, variations in $r_{\mathrm{MRR}-a}$ exert a substantial influence on both the insertion loss and extinction ratio. A transmittance exceeding 80% is achieved when $r_{\mathrm{MRR}-a}$ remains within the range of 4994.2 nm to 5006.4 nm. Beyond this interval, increased insertion loss occurs due to misalignment of the primary resonant peaks among MRR-a, MRR-b, and MRR-c. Thus, the fabrication tolerance for the radius $r_{\mathrm{MRR}-a}$ is ±6.1 nm, corresponding to a total diameter variation of 24.4 nm.
(3) To evaluate the impact of temperature fluctuations (t) on device performance, simulations were conducted using a temperature step size of 0.8 K. The insertion loss across a temperature range of 280 K to 320 K was computed employing the “DEVICE” and “Interconnect” modules in Lumerical. As shown in Figure 12, insertion loss is sensitive to temperature variation because fluctuations alter the effective refractive index of silicon, leading to a shift in the resonant peak. This disrupts the resonant condition and prevents effective confinement of light at the operational wavelength. Consequently, while transmittance remains above 80% within the interval from 294 K to 306 K, large temperature variations significantly increase insertion loss. Thus, the operational temperature tolerance of the proposed device is 294 K to 306 K, corresponding to a range of 12 K.

## 5. Discussion

Table 2 provides a performance comparison between conventional manual designs and our DBS-optimized triple-ring filter. Manual design methods [14,24,25,26,27] often result in widely varying performance: while some achieve low insertion loss (e.g., 0.36 dB in [25]), others suffer from high loss (e.g., 18.5 dB in [26]) or limited extinction ratio (e.g., 5.8 dB in [26]). Trade-offs are also evident, such as the sacrifice of FSR for narrow linewidth, as in [27], where a 9 nm FSR was accompanied by 5.6 dB insertion loss. Inconsistent performance across manual designs underscores their dependence on designer intuition and limited parameter exploration. Furthermore, as we can see in Table 2, manual designs typically exhibit higher insertion loss since they consider limited structural parameters while overlooking critical details like coupling efficiency. Conversely, as shown in Table 2, devices assisted with the DBS algorithm consistently achieve low insertion loss by continuously iterating nanostructures, which simultaneously reduces losses and maintains a relatively large extinction and FSR.

As shown in Table 2, the footprints of the proposed device are larger than those of multimode interference (MMI) structures designed using the DBS algorithm. This is primarily because implementing the Vernier effect requires slightly different ring circumferences, while avoiding excessively small dimensions is necessary to maintain resonant peak density. Furthermore, although MMI couplers can be designed with very compact footprints [17,18,19,20], they exhibit inherent limitations such as relatively broad output linewidth and comparatively small FSR. However, enlarging the device dimensions also increases transmission loss, which poses a fundamental limitation for the proposed triple-ring optical filter. Therefore, we utilized TCMT to reveal how its light transmission efficiency depends on the coupling attenuation coefficient between rings and waveguides. Then, we focused on the design of the coupling structure between the rings and the waveguides, simultaneously employing the DBS algorithm to minimize insertion loss. Furthermore, a comprehensive comparative analysis was conducted between the theoretical DBS algorithm and the manually designed device transmission spectra by modeling the triple-ring optical filter using the 3D-FDTD method. The numerical results show that the proposed device exhibits insertion loss, extinction ratio, FSR, and linewidth of 0.4 dB, 20 dB, 86 nm, and 0.2 nm, respectively, which is superior to most of the current studies presented in Table 2.

It is also important to note that the performance of the current design is limited by the material properties of the silicon waveguides. Employing low-loss materials such as Si_3_N_4_ could further reduce propagation loss and potentially improve overall device performance. Based on our current findings, some promising research directions and applications can be proposed: First, substituting the third ring with a higher-Q resonator could achieve a narrower linewidth. Second, implementing this cascaded structure as a laser external cavity would directly compress output linewidths by extending cavity lengths for coherent optical communications. Third, the device’s narrow linewidth, high extinction ratio, and large FSR enable resonant wavelength tuning via integrated heaters for DWDM. Finally, the proposed triple-ring filter also shows strong potential for high-performance on-chip optical sensing [28]. In particular, its narrow linewidth, which results in higher spectral sensitivity, combined with a large FSR, makes it suitable for high-sensitivity sensing compatible with broad measurement ranges [29]. The Vernier effect, leveraged in this design, can further enhance sensitivity in cascaded resonator-based sensors [30].

## 6. Conclusions

This study established a TCMT-based theoretical model for triple-ring cascaded structures, deriving a maximum transmittance of 1. Then, we identify the coupling efficiency as the core determinant of transmission performance in triple-ring filters, leading us to establish an FOM for DBS algorithm-driven coupling structure optimization. Using 3D-FDTD simulations, we validate device performance, achieving 0.4 dB insertion loss, 20 dB extinction ratio, and 86 nm FSR within a compact 29 μm×46.5 μm footprint featuring 0.2 nm linewidth. The implemented DBS algorithm enhances transmission performance compared to conventional designs. Our design methodology, which leverages the DBS algorithm, offers an efficient approach for developing complex photonic devices. It is directly applicable as a laser external cavity for coherent optical communications and also shows significant potential for deployment in DWDM systems and sensing applications.

## Figures and Tables

**Table 1:** Structural parameters.

**Parameter**	**Value**	Parameter	Value
$R_{1}$	5 μm	$W_{wg}$	0.45 μm
$R_{2}$	5.9 μm	$H_{wg}$	0.22 μm
$R_{3}$	9.7 μm	$W_{MRR}$	0.45 μm
d	13 μm	$H_{MRR}$	0.22 μm
D	4 μm	Size	29 μm×46.5 μm

**Table 2:** Performance Comparison.

References	Method	Insertion Loss (dB)	Extinction Ratio (dB)	FSR (nm)	Foot Print (μm^2^)	Line Width (nm)
[14]	Manual	1	5.78	72	-	0.62
[17]	DBS	0.86	16.8	70	42×6.8	-
[18]	DBS	0.759	10.06	50	2.4×3.6	-
[19]	DBS	0.5	20	40	3×4	-
[20]	DBS	0.82	18.1	35	3.6×2.4	2
[24]	Manual	2.2	11.5	35	475	0.4
[25]	Manual	0.36	25.07	7.8	23.8×20.82	0.5
[26]	Manual	18.5	5.8	15.76	-	10
[27]	Manual	5.6	13	9	-	1
This work	DBS	0.4	20	86	29×46.5	0.2

## Data Availability

No new data were created in the present research.
